# Revisiting clinical trials on glycemic control and cardiovascular risk

**DOI:** 10.1186/1758-5996-1-12

**Published:** 2009-09-28

**Authors:** Sandra Roberta Gouvea Ferreira

**Affiliations:** 1 Nutrition Department, School of Public Health, University of São Paulo, Brazil

## Abstract

The most relevant clinical trials, assessing the role of glycemic control in reducing cardiovascular risk, are examined. The UKPDS was the first to address this issue. More recent trials (ACCORD, ADVANCE and VADT) are controversial and evidences did not support that strict glycemic control (reflected by normal glycated hemoglobin) exclusively is sufficient to reduce cardiovascular risk in complicated individuals with long-term type 2 diabetes mellitus. Some possible reasons for controversies are included.

## Commentary

In the last years, an increase in the cardiovascular mortality of diabetic individuals has been observed, being more significant among diabetic women [1]. The presence of diabetes mellitus has a deleterious effect on mortality independently of other risk factors, resulting in a two-fold increase in the mortality rate [2]. Recently, Haffner et al [3] observed that diabetes is considered an equivalent of high cardiovascular risk, as diabetic individuals, even without a previous cardiovascular event, present an incidence of myocardial infarction in a period of seven years similar to non-diabetic individuals who have already presented cardiovascular events.

Since the 1960s, several clinical trials evaluated the role of glycemic control in decreasing cardiovascular risk. However, by the end of the 1990s and especially in the beginning of this century, the main clinical studies focusing on the role of the glycemic control for the decrease of cardiovascular risk were conducted, such as the UKPDS - United Kingdom Prospective Diabetes Study [4] and Steno-2 [5]. In this review, we will emphasize mainly the three latest studies: the ACCORD - Action to Control Cardiovascular Risk in Diabetes [6], ADVANCE - Action in Diabetes and Vascular Disease - Preterax and Diamicron Modified Release Controlled Evaluation [7] and VADT - Veterans Affair Diabetes Trial [8].

The UKPDS [4] was the first of these studies and it was a milestone in diabetology. The investigators observed that individuals submitted to intensive glycemic control had a risk reduction of 16%, which was not significant with regards to macrovascular events, especially acute myocardial infarction and sudden death. UKPDS also found that a 1% decrease in glycated hemoglobin reduced 21% of the risk of any diabetes-related outcome, 14% the risk of myocardial infarction and 37% the risk of microangiopathy [9]. Steno-2 [5] which was a study of intensive intervention in multiple cardiovascular risk factors in type 2 diabetic individuals with microalbuminuria, showed that in addition to the intensive glycemic control, the control of other risk factors frequently observed in these individuals such as hypertension, dyslipidemia and microalbuminuria, significantly decreased the risk of cardiovascular events and microangiopathy. The major conclusion of Steno-2 was that the exclusive control of glycemia is not effective for the cardiovascular protection in type 2 diabetes, since a number of other risk factors - which constitute the metabolic syndrome - must be also intensively treated. Steno-2 has recently reported the results of the further 5-year continuation, after eight years of follow-up with interventions, in a total of 13 years of follow-up [10]. The results showed that the multifactorial intensive therapy continued to be associated with significant benefits on the cardiovascular system, with an even more significant decrease regarding cardiovascular events and death than during the initial intervention period. Should we control glycemic levels? We actually have enough evidence to affirm so, but in addition to controlling glycemia very strictly, we should also control blood pressure levels, lipid profile and other risk factors. However, it is important to emphasize that the decrease in glycated hemoglobin (HbA1c) while reducing the risk of chronic complications increases the risk of hypoglycemia, which is a frequent adverse effect during intensive control of glucose levels.

Meta-analysis has already demonstrated an independent association between the glycemic control and the incidence of cardiovascular disease in type 1 and type 2 diabetes [11]. In type 1 diabetic individuals [12], it has been observed that the increase in HbA1c was associated with a significant increment in the incidence of peripheral arterial disease, and a borderline significant increase in the incidence of coronary heart disease, even after adjustment for a number of risk factors. The conclusion is that there are evidences of association between HbA1c reduction and cardiovascular benefits in diabetes. However, up to which level of HbA1c reduction would there be benefits to the patient? Would the established goal of HbA1c reduce the incidence of cardiovascular events? Randomized clinical trials, particularly with type 2 diabetes individuals, were necessary to answer this question.

To answer this question properly, clinical trials should have several strengths. In addition to the clearness of this question, the design should be longitudinal and long enough to allow the occurrence of outcomes. Also, the sample size is important as well as being randomized to minimize bias of several natures. Taking hard outcomes rather than surrogate outcomes is preferable, and statistical analysis should consider adjustments for a number of confounding variables. Trials attending these characteristics should generate high quality evidences. Fulfilling most of these aspects, two studies were conducted and recently the ACCORD [6] and ADVANCE [7], and additionally the VADT [8] results were announced in the 2008 Scientific Sessions of the American Diabetes Association. These three trials were designed specifically to investigate whether a more strict goal of glycated hemoglobin would further reduce the risk of cardiovascular events in type 2 diabetes than the currently recommended goal, which is HbA1c < 7%.

ACCORD was a study conducted in North America, with the participation of over ten thousand patients who had initial HbA1c > 7.5% and high risk of cardiovascular disease. The hypothesis of this arm of the ACCORD - referring to glycemic control - was that reduction of HbA1c to less than 6% would bring more benefits with regards to macrovascular events. An important proportion of the patients in this study already had a cardiovascular event. The subset allocated to the intensive glycemic control had the goal of HbA1c < 6% while the control group had the goal of between 7.0 and 7.9%.

The primary outcome was myocardial infarction, non-fatal stroke and cardiovascular death; the follow-up was initially scheduled to 5.6 years, but the glycemic arm was interrupted prematurely with 3.6 years due to the report of significant excess of mortality in the intensive group, in which 257 deaths occurred, compared with 203 in the control group.

At the beginning of the study, these individuals had a mean age of 62 years, 35% had history of a previous cardiovascular event, and mean value of HbA1c was 8.3% for both groups. These were also comparable with regards to the other variables. By the end of the follow-up period, individuals undergoing intensive therapy presented a significantly higher frequency of use of antidiabetic agents, especially metformin, secretagogues and glitazone when compared with those under standard therapy. This resulted in HbA1c of 6.4% in the intensive group and of 7.5% in the conventional therapy group. It is important to observe that the dropping rate of HbA1c was very fast in the intensive therapy group. The individuals under intensive therapy presented higher exposure to anti-hyperglycemic drugs, had more hypoglycemia, more weight gain and water retention, and they also used ACE inhibitors less often.

Regarding the primary outcomes of the glycemia arm of the ACCORD study [6], there was no statistically significant difference between the individuals under standard therapy and those under intensive therapy, and the latter presented significantly higher mortality when compared to patients under standard therapy. However, an analysis of a sub-group Eoof individuals included in the ACCORD who had not had previous cardiovascular disease showed that these individuals also reach benefits from the intensive treatment. The patients who presented lower HbA1c at the beginning of the study apparently also had benefits from the intensive therapy.

The most frequent adverse effect in the ACCORD [6] group under intensive therapy, as expected, was hypoglycemia and especially severe hypoglycemia. This group also presented higher frequency of water retention and weight gain > 10 kg in 28% of the patients enrolled. Summing up, the main findings of ACCORD, in type 2 diabetes individuals with high cardiovascular risk and initial HbA1c > 7.5%, the therapy seeking a more strict glycemic control, i.e., HbA1c < 6% in 3.6 years, increased all-cause mortality rates, in addition to a higher weight gain and more hypoglycemic events. Considering the combination of primary outcomes, the ACCORD does not support the hypothesis of benefit from intensive glycemic control to the reduction of cardiovascular events in patients with high cardiovascular risk.

The ACCORD [6] had several favorable aspects such as hard outcomes, the sample size (involving a high number of participants) and the statistical analyses were carefully performed. Among the unfavorable aspects of the study, we mention the selection of patients with high cardiovascular risk, which possibly do not represent most of the patients in our offices and outpatient clinics, limiting to generalize these results to the whole population of diabetics. Other unfavorable aspects were the multiple possibilities of drug interactions and the follow-up of 3.6 years, which may be considered relatively short to assess hard cardiovascular outcomes. The possible explanations argued for these unexpected ACCORD results could be related to drugs such as rosiglitazone or deleterious drug interactions. Apparently, this was not the case, because in the analysis involving the subgroup of patients who used rosiglitazone, no higher occurrence of these undesirable effects was observed. The higher frequency of hypoglycemia should be related to the multiple insulin doses or to the aggressive and/or fast approach to achieve the glycemic goal (HbA1c level < 6% was low).

The ADVANCE study [7] was conducted in several countries, with over 11 thousand patients aged over 55 years old. Similarly to the ACCORD [6], its objective was to assess the reduction of combined micro and macrovascular events, in type 2 diabetes patients with history of micro- or macrovascular disease or presenting risk factors, through intensive glycemic control, under a regimen based on sulfonylurea, as initially all the individuals used gliclazide. The study had HbA1c of 6.5% as a goal, intensive blood pressure control with a fixed combination of ACE inhibitor plus a diuretic. The study was divided into intensive or standard glycemic control, and these two arms were subdivided into two treatment groups for hypertension, intensive and standard groups. This study also had as primary outcomes macrovascular disease, which would be the combination of stroke, non-fatal infarction and cardiovascular death, but it also included microvascular events, defined as the appearance or worsening of retinopathy and nephropathy. Both groups presented, at the beginning of the study, mean HbA1c of 7.5%, and its decrease in the intensive group up to the goal of 6.5% was slower in the first months, than observed in the ACCORD study [6]. Regarding the fasting glycemia, the behavior was similar. Favorable results regarding the primary outcome - combining macro- and microvascular events - were statistically significant in the intensive treatment group, but when we separate macro- from microvascular events, we observe that the decrease in microvascular events was remarkable, but not in macrovascular events. The frequency of hypoglycemia was higher in the intensive treatment group, but the frequency of severe hypoglycemia in this study was very lower than that observed in the ACCORD study [6].

As main findings of this study, we would say that the intensive glycemic control resulted in a 10% decrease in the composite outcome, 14% decrease in microvascular events, 21% in nephropathy, but without significant effect regarding macrovascular events and cardiovascular mortality. Regarding the reduction of microangiopathy, previous studies, such as UKPDS [4], had already shown that the intensive control resulted in significant improvement. However, regarding the absence of effects at least deleterious on the cardiovascular system, it was different from the already shown findings of the ACCORD [6]. These two studies present similarities: as they had no conflicts of interest with private companies, sample sizes were similar and expressive, both presented high power to detect the risk decrease, both analyzed data by intention to treat technique, HbA1c at baseline in the standard and intensive groups were similar, one third of the patients in both studies presented previous family history of cardiovascular disease. However, they presented some differences: in the ACCORD, individuals were older; baseline HbA1c was lower in the ADVANCE [7] when compared to the ACCORD; the glycated hemoglobin goals were lower in the ACCORD, 6% as compared to the ADVANCE, which was 6.5%, and the rate to achieve these goals were also different from one study to another. For instance, in the ACCORD, there was a 1.4% decrease in HbA1c in the first four months, while in the ADVANCE, HbA1c decreased 0.5% in the first six months and further 4.4% in the following six months. The primary outcomes were also different: in the ACCORD, it was purely cardiovascular and in the ADVANCE study, it was a combination of micro- and macrovascular outcomes, and death was a secondary outcome in the ADVANCE.

Possible reasons for these contrasting results are: outcomes were not comparable, different frequencies and drug types were used, e.g. when it comes to glitazones, in the ADVANCE, less than 20% of patients were under such therapy, and in the ACCORD, over 90% of patients in intensive therapy were using the drug. The follow-up period was also shorter in the ACCORD study when compared to 5 years in the ADVANCE, hypoglycemia was more frequent and there was considerable weight gain, 3.5 kg on average, only in the ACCORD, specifically in the group of intensive therapy.

The VADT [8] was the third large study of tight blood glucose control, in which war veterans from 20 Veterans' Association Hospitals in U.S. were enrolled. Patients with poorly controlled type 2 diabetes were randomized to either intensive glucose control or standard therapy. The goal was a HbA1c value < 7% for the group under intensive therapy and 8-9% for the group under standard therapy and the primary outcome of the study were macrovascular complications.

VADT [8] included a much smaller sample (n = 1,791) than the studies previously mentioned, aged over 60 years old, body mass index compatible with obesity, with uncontrolled diabetes using one or more oral agents or insulin, following an algorhythm which started the treatment with metformin for obese individuals or sulfonylurea for non-obese patients, associating gradually other oral agents, including insulin. The individuals had a known period of diabetes diagnosis of 12 years and they had already presented a very elevated frequency of chronic complications of diabetes, and hemoglobin at the beginning of the study was 9.4%. The study length was 7.6 years and the primary outcomes were only cardiovascular events (myocardial infarction, death, stroke, cardiac failure, amputation due to peripheral arterial disease, surgical intervention or angioplasty and critical ischemia in limbs).

Individuals were stratified into two types of treatment: intensive and standard, the number of individuals who presented events in the groups was not different, the average of HbA1c after six years of follow-up in the standard group was around 8.4%, while in the group under intensive treatment, it was 6.9%. The conclusion of the VADT investigators was that in an advanced age population, with long-term diabetes and chronic complications, it is more difficult to manage the therapy, but it is possible to obtain significant improvement in glycemic levels and in the other risk factors of these individuals; however, isolated glycemic control does not have significant effect in reducing cardiovascular events.

Considering that the rates of cardiovascular events found in these studies were lower than the previously described ones, we may infer that the modern therapies for type 2 diabetes have significantly benefits to these patients. It is necessary to establish the level of glycemic control which will be favorable to the patient, either with high or low cardiovascular risk, as well as to define the ideal rate to achieve such control level. Finally, the effects of drug combinations should also be investigated.

## Competing interests

The author declares that they have no competing interests.

## Authors' contributions

SRGF reviewed the literature and made the commentary on the issue.
